# Targeted molecular CT imaging of atherosclerosis and abdominal aortic aneurysm

**DOI:** 10.7150/thno.137688

**Published:** 2026-08-24

**Authors:** Jannis Marchand, Felix Stefan Reuter, Hannah Lichtmannegger, Keno Kyrill Bressem, Lisa Christine Adams, Franz Pfeiffer, Avan Kader, Marcus Richard Makowski

**Affiliations:** 1 Technical University of Munich, Institute of Diagnostic and Interventional Radiology, Ismaninger Straße 22, 81675 Munich, Germany.; 2 Technical University of Munich, Institute of Cardiovascular Radiology and Nuclear Medicine, Lazarettstraße 36, 80636 Munich, Germany.; 3 Technical University of Munich, Munich Institute of Biomedical Engineering, Boltzmannstraße 11, 85748 Garching, Germany.; 4 Technical University of Munich, Chair of Biomedical Physics, Department of Physics, School of Natural Sciences, Paula-Hahn-Weinheimer-Straße 1, 85748 Garching, Germany.; 5 Technical University of Munich, Institute for Advanced Study, Lichtenbergstraße 2a, 85748 Garching, Germany.

**Keywords:** molecular imaging, computed tomography (CT), abdominal aortic aneurysm (AAA), atherosclerosis, targeted contrast agents

## Abstract

Atherosclerosis and abdominal aortic aneurysm (AAA) share the same pathological triad, which includes chronic inflammation, immune cell infiltration, and extracellular matrix (ECM) degradation. Both diseases are characterized by the fact that the disease process may not be diagnosed until a life-threatening event occurs. Current imaging methods diagnose these conditions by assessing the degree of stenosis of the blood vessel, the plaque burden, and the aneurysm diameter. Unfortunately, these methods are not sufficient to measure biological activity, which is important to determine if such potentially life-threatening events will occur and where they will occur. Molecular imaging, together with computed tomography (CT), might close this gap. CT offers several benefits: it is widely available, allows rapid acquisition, can be integrated into vascular workflows, and can provide quantitative signal readout, but its main use has been in the context of anatomical detail. Recent preclinical studies suggest that a shift may be occurring in this area. This review examines the use of molecular CT imaging for the diagnosis of atherosclerosis and AAA, systematically grouped by molecular target. There are three different approaches that are employed in the current literature. The most clinically advanced is the first, macrophage-targeted probes. The second, high-density lipoprotein (HDL)-mimetic nanoparticles, takes advantage of an endogenous lipoprotein pathway, but not an engineered ligand. The third, gold nanoparticles, which targeted elastin, yielded the most clinically relevant finding to date: *ex vivo* signal correlates inversely with aortic wall burst pressure, casting doubt on whether diameter is sufficient to assess for rupture. Comparable methods for targeting biomarkers with CT have not been developed, although magnetic resonance imaging (MRI) and nuclear imaging modalities can already target matrix metalloproteinase (MMP) activity, vascular cell adhesion molecule-1 (VCAM-1), and microcalcification. This is a space that could be very fruitful for future studies.

## Introduction

Cardiovascular diseases (CVD) are a collection of diseases of the heart and blood vessels. They remain one of the main causes of death around the world 1. Around 19.8 million people died from CVD in 2022, accounting for around 32% of all deaths. Over three-quarters of these were in low- and middle-income countries 2. Atherosclerosis is a long-term inflammatory disease of large and medium arteries, defined by lipid accumulation and plaque formation 3, 4. AAA is caused by progressive degeneration of the aortic wall and abnormal remodeling of the extracellular matrix. These two conditions are among the most important types of arterial disease in clinical practice 5 and progress quietly over many years 6, 7. Atherosclerosis may cause heart attacks, strokes, or arterial blockages in the limbs, whereas AAA can result in aortic rupture 5, 8, 9. Each of these outcomes constitutes a life-threatening event.

Acute cardiovascular events often originate from atherosclerotic lesions that do not result in hemodynamically significant stenosis 10, 11. In AAA, the biological mechanisms leading to wall failure progress independently of aneurysm diameter 12. Therefore, diameter can only be used as a limited surrogate for rupture risk 13. In both, the anatomy of the vessel is not a representative indicator of disease activity. Anatomical imaging alone cannot fully reveal important biological processes such as chronic inflammation, ECM breakdown, and active microcalcification 14, 15. This has led to an increasing interest in imaging biomarkers reflecting vessel wall biology rather than just its structure. Early detection of these changes is important for timely, biology-based treatment before they become a major event.

CT has distinct advantages over other imaging modalities: it is widely available, often used in clinics, and provides detailed, quantitative images of vascular wall structures. These strengths make it a good choice for translational molecular imaging. This review discusses current targeted CT contrast agents and emerging molecular CT imaging techniques for atherosclerosis and AAA. It also reviews current preclinical evidence and its potential for translation to the biological characterization of the vessel wall.

## Structural organization of the arterial wall

### The arterial system

The arterial system serves as the principal conduit for blood distribution and is integral to the development of cardiovascular diseases. Age-related and pathological changes, such as increased wall stiffness, endothelial dysfunction, and progressive loss of elasticity, increase the arterial wall's susceptibility to damage 16. Understanding the structural organization of the arterial wall provides a basis for understanding arterial pathologies, including atherosclerosis and AAA. The arterial system consists of large and medium arteries, which are structurally and functionally distinct vessel types that jointly enable blood transport, pressure regulation, and tissue perfusion 17.

Arteries facilitate the transport of blood from the heart to other parts of the body. They are divided into elastic and muscular arteries. Elastic arteries, such as the aorta and other large vessels, possess an elastin-rich medial layer that enables them to buffer pulsatile pressure and sustain continuous downstream blood flow 18, 19. This ability, called the Windkessel function, reduces the heart's workload and smooths out blood flow in smaller arteries further from the heart 18. Muscular arteries, such as the femoral, radial, and coronary arteries, are distinguished by a higher proportion of vascular smooth muscle cells (VSMCs), which allow active regulation of blood flow to specific organs (Figure 1) 20, 21.

Arterioles, also called resistance vessels, are small arteries that connect muscular arteries to capillaries. They regulate systemic vascular resistance and local blood flow by coordinating endothelial cell and VSMC function 22, 23. Capillaries are the smallest blood vessels and consist solely of endothelium and a basement membrane. They link the arteries and veins. Through their thin walls, they facilitate the exchange of gases, nutrients, and metabolic by-products between blood and tissue 24, 25.

All arteries, despite their different functions, have the same basic three-layered wall structure: the *tunica intima*, *tunica media*, and *tunica adventitia* (Figure 1). These layers collectively confer mechanical stability and elasticity while continuing vascular homeostasis 26-28. Table 1 summarizes the composition and function of each layer.

The ECM plays a major role in determining the mechanical properties of the arterial wall. Its composition and turnover are key to maintaining wall stability and supporting remodeling 35, 36.

### The extracellular matrix

The ECM is primarily composed of fibrous proteins, glycoproteins, and proteoglycans in a three-dimensional scaffold. The ECM is the structural framework for the vessel wall and plays a major role in the mechanical properties of the wall, and the composition and turnover of the ECM are important in regulating aspects of cellular behavior 35-38. The VSMCs are the predominant source of ECM components in the *tunica media* of the arterial wall, while the *tunica adventitia* is the source of matrix production by the fibroblasts 39.

Elastin is the main component of elastic fibers and the reason for maintaining the flexibility of large arteries. Elastin is arranged in concentric elastic lamellae, which allow the conducting arteries, such as the aorta, to expand during systole and contract during diastole. This process dampens fluctuations in blood pressure and ensures a steady flow of blood. A gradual loss or breakdown of elastin makes arteries less flexible and weakens their walls 18.

The collagen fibers are primarily type I and III, and they provide strength and reduce excessive distention of the vessels under hemodynamic stress. Impaired collagen production or breakdown leads to pathological remodeling and a weakened vessel wall 35.

Although fibrin is not a permanent component of the ECM, it is an important part of the process of repairing blood vessels. Once endothelial cells have been disrupted, thrombin converts fibrinogen to fibrin monomers that polymerize to create a provisional thrombus. Factor XIIIa cross-links fibrin polymers, forming a mechanically stable structure that is important for wound sealing and cell migration 40-42.

Proteoglycans and glycosaminoglycans control matrix organization and cell signaling through their ability to bind cytokines, growth factors, and lipoproteins 43-45. They have high water binding capacity that helps to maintain matrix hydration 46 and binding and migration to cell surface receptors 38.

### Matrix remodeling

The ECM is maintained in a dynamic state of balance between ongoing synthesis and proteolysis. The MMPs are zinc-dependent enzymes that mainly degrade collagen and elastin, and other ECM components 39. Tissue inhibitors of metalloproteinases (TIMP) regulate MMP activity, resulting in a controlled balance in healthy tissue 47, 48. An imbalance of this can lead to reduced arterial wall stability, either because of increased MMP activity or because of changes in TIMP levels. These alterations promote the destabilization of atherosclerotic plaques and the progression of AAA 49.

## Pathophysiology and clinical imaging of atherosclerosis and AAA

### Atherosclerosis

Atherosclerosis is a chronic inflammatory disease of large and medium-sized arteries. It is defined as the development of lipid-rich plaques in the wall of the vessel, and is the principal pathological substrate of several major CVDs 50-53. Endothelial dysfunction is a primary event in the initiation of the disease, whereby the endothelial barrier and regulatory functions of the arterial wall are impaired 54. In addition to these, elevated low-density lipoprotein (LDL) cholesterol 55 and modifiable risk factors such as high blood pressure, diabetes, obesity, and smoking 50, 56, and non-modifiable risk factors such as age, sex, and genetic predisposition play a role in disease development 50, 57, 58. The key cellular mechanisms and stages of atherosclerotic progression are summarized in Figure 2.

Impaired endothelial permeability, likely caused by endothelial dysfunction, allows infiltration of LDL into the *intima* and its oxidation into oxidized LDL (oxLDL) 54. OxLDL induces pro-inflammatory responses by activating endothelial cells and upregulates the expression of adhesion molecules such as VCAM-1 and intercellular adhesion molecule-1 (ICAM-1). VCAM-1 and ICAM-1 promote the recruitment and transmigration of circulating monocytes into the vessel wall 56. Once within the *intima*, monocytes differentiate into macrophages, which internalize oxLDL and transform into lipid-laden foam cells. This sequence defines the early stage of fatty streak formation, a process that can begin during childhood 7, 58, 62.

Foam cells and oxLDL sustain local inflammation and facilitate the recruitment of additional immune cells 63-65. Defective efferocytosis, the process by which apoptotic cells are cleared by phagocytes, results in the accumulation of extracellular lipids, cellular debris, and cholesterol crystals, which promotes necrotic core formation 66. Macrophages with a pro-inflammatory M1 phenotype release cytokines such as tumor necrosis factor-α (TNF-α) and interleukin-6 (IL-6), consequently sustaining vascular inflammation as well as promoting lesion progression. In contrast, anti-inflammatory M2 macrophages are more abundant in stable plaques and are linked to tissue repair and lesion regression 67. VSMCs migrate into the inner layer of the artery, proliferate, and produce components of the ECM 63, 68. This leads to the formation of a fibrous cap, mostly collagen, that covers the fatty core of the plaque 68, 69.

Lesion progression results in ongoing inflammatory activity, which leads to ECM remodeling inside the plaque. MMPs derived from the macrophages break down collagen in the fibrous cap, resulting in a progressive reduction in cap thickness and tensile strength 70, 71. A thin or weakened fibrous cap is a feature of vulnerable plaques and a risk factor for plaque rupture 69, 72.

Vascular calcification occurs at the same time. This is achieved by passive calcium-phosphate deposition as well as by the activation of osteogenic differentiation of smooth muscle cells 73, 74. The pattern matters clinically. Microcalcifications in the fibrous cap are thought to be a feature that creates mechanical stress, leading to plaque rupture, while macrocalcification is thought to be a stabilizing feature 73, 75. Once a plaque is unstable or ruptures, the thrombogenic material is exposed to the blood. This exposure can lead to the formation of a thrombus that can cause myocardial infarction, stroke, or peripheral ischemia, depending on the vessel involved 76, 77.

The phenotype of plaques is determined by a combination of morphological features such as necrotic core size, fibrous cap integrity, and calcification pattern, and has a significant effect on the rupture risk. Lesions that lead to the development of acute cardiovascular events, like myocardial infarction and ischemic stroke, are often without the presence of a hemodynamically significant stenosis 10, 11. Therefore, identifying biologically active, rupture-prone plaques, regardless of stenosis severity, remains a major unresolved challenge in the clinical management of atherosclerotic disease.

### Abdominal aortic aneurysms

AAA is defined as the localized and sustained dilation of the abdominal aorta with changes in the wall structure. AAA is one of the most common types of aneurysms 78. It affects about 4% of men and 2% of women over 65 years old, and if it ruptures, the risk of death is over 80% 78-82. Diagnostically, an aneurysm is defined as a permanent localized dilation of an artery with an increase in diameter of at least 50% compared to the expected normal diameter of the artery in question 83 or, in practice, as an absolute diameter of ≥ 30 mm 84. Because the course of AAA is often so asymptomatic, the condition is often found later in the course. At present, there is no pharmacologic therapy that can slow the growth of an aneurysm or decrease the risk of an aneurysm rupturing 85, 86.

When detected, the AAA are categorized based on their size as small (< 55 mm) or large (≥ 55 mm) 87. Treatment of small aneurysms is generally aimed towards watchful waiting with periodic imaging surveillance. On the other hand, those aneurysms that are more than 55 mm are treated surgically, due to the high risk of rupture. Female patients will often have lower thresholds of intervention 88, 89. Intervention is the main therapeutic approach, with improvements in endovascular and surgical procedures in high-risk AAAs 90.

While there are similarities in risk factors associated with both AAA and atherosclerosis (such as age, male gender, smoking, hypertension, and family history), the pathogenesis is quite distinct 91. Atherosclerosis involves mostly the degradation of the ECM of plaques in the *intima*, while in AAA tissue remodeling and abnormalities occur throughout the aortic wall 92-94. Furthermore, diabetes mellitus, a well-established risk factor for atherosclerosis, is associated with a lower risk of AAA 95, 96, which further suggests that these conditions are pathobiologically different entities 97. Notably, several studies have reported an association between metformin use and both lower AAA incidence and reduced growth rate 98, 99.

Three closely interconnected processes are involved in the development of AAA – chronic inflammation, loss of VSMCs, and ECM degradation – 93, 100-102. A series of such mechanisms is dynamic, interacting intimately and reinforcing each other in an order not predetermined but rather emergent 103.

No single cause has been established, but tobacco smoke is the most significant modifiable risk factor for AAA; whereas the presence of atherosclerotic lesions and hypercholesterolemia might play a role in the development of AAA, but do not necessarily trigger it 78, 104.

Early stages of AAA are associated with inflammation, which is typically characterized by elastin degradation, representing one of the initial events in ECM remodeling 105. Aortic wall compliance is significantly reduced at the time of loss of elastin and sets off a self-propagating inflammatory cascade 106. Activated VSMCs increase inflammation by producing increased levels of pro-inflammatory mediators. Elastin degradation products also attract immune cells from the blood to enhance local inflammation 91. Immune cells are recruited as a result of inflammatory chemokines and cytokines, which allow for infiltration of leukocytic cells, such as monocytes, into the wall of the aneurysm 107. Continued accumulation of monocyte-derived macrophages and other leukocytes increases protease activity, mainly through the upregulation of MMP expression, thus promoting ECM degradation and aortic wall weakening 100. These immune cells produce pro-inflammatory cytokines such as interleukin-1β (IL-1β), IL-6, and TNF-α, sustaining inflammation and recruiting further immune cells 108. Although the pro-inflammatory M1 macrophage state is a hallmark of the progression of AAA, M2 macrophages have anti-inflammatory and tissue-repairing properties. The ratio of M1 and M2 macrophages in the aneurysmal wall is increasingly recognized as an important factor of disease progression 109. In addition, chronic inflammation leads to neovascularization of the medial walls, which allows further infiltration of immune cells and helps sustain the inflammatory milieu 100.

At the same time, the process of oxidative stress in the wall of the aneurysm causes a rise in proteolytic activity, which results in apoptosis of endothelial cells and VSMCs 110. As the disease progresses, other immune cell types, including T lymphocytes and neutrophils, also contribute to raising the inflammatory response 111.

Both conditions show the same imbalance in matrix turnover: reduced TIMP levels alongside elevated MMP-2 and MMP-9, resulting in accelerated ECM degradation 112, 113. The most important pathological mechanisms contributing to AAA disease progression are depicted in Figure 3 and include inflammation and ECM degradation.

Importantly, the biological processes that underlie the development of aneurysms in atherosclerosis are spatially and temporally variable and do not depend on aneurysm size 12. The biological nature of aortic wall failure makes the AAA more aptly described as a disease of progressive inflammation than any disease of aortic diameter, and diameter-based criteria are a limited surrogate for biological disease activity and for aortic wall rupture risk 13.

### Clinical imaging of atherosclerosis and AAA

Non-invasive imaging is key for diagnosing, monitoring, and assessing the risk of atherosclerosis and AAA 116, 117. Clinical modalities include ultrasound (US), MRI, CT, and nuclear medicine techniques like positron emission tomography (PET) and single-photon emission computed tomography (SPECT) 118, 119. There are differences in the capabilities of each method for anatomical detail, soft tissue imaging, molecular sensitivity, and clinical utility 117.

In clinical practice, US is widely used as the primary imaging method as its inexpensive, non-invasive, and easily accessible. It is the recommended technique for AAA screening and surveillance and is often used to determine the severity of carotid artery stenosis 116, 120. For comprehensive anatomical assessment and pre-interventional planning, computed tomography angiography (CTA) is the modality of choice and the gold standard for preoperative imaging of AAA 88, 89, and is increasingly used in the initial evaluation of atherosclerotic disease when intervention is considered 121, 122. The high spatial resolution and rapid image acquisition of CTA allow for detailed analysis of stenosis severity, plaque burden, calcification patterns, aneurysm diameter, branch vessel anatomy, and intraluminal thrombus 117. Furthermore, CTA can characterize plaque vulnerability by identifying high-risk morphological features, including low-attenuation plaque, positive remodeling, spotty calcification, and napkin-ring sign, which are all associated with adverse cardiovascular outcomes 123.

MRI has excellent soft-tissue contrast and provides detailed information about the composition of the vessel wall and plaque, such as intraplaque hemorrhage and lipid-rich necrotic core 124. PET – and, to a lesser extent, SPECT – provides highly sensitive detection of molecular disease activity, including vascular inflammation and active microcalcification 125, 126, but remains limited to selected clinical indications and research settings due to restricted availability, higher costs, and limited spatial resolution 126, 127.

Anatomical imaging alone cannot capture the biological processes that characterize both conditions, including chronic inflammation, ECM degradation, and active microcalcification 14, 15. Consequently, research interest has shifted toward imaging biomarkers that directly reflect these biological processes. CT is a plausible candidate for this purpose. CT is widely available, provides inherently quantitative signals, and offers sufficient spatial resolution to resolve the vessel wall.

## Nanoparticle-based CT contrast agents by molecular target

To resolve these limitations, targeted nanoparticle-based contrast agents have been developed to extend CT beyond anatomical assessment toward the visualization of disease-associated biological processes, including inflammatory cell infiltration, ECM remodeling, and microcalcification. Current preclinical evidence for these strategies is presented by molecular target, rather than by disease entity. This structure underlines common pathobiological mechanisms of atherosclerosis and AAA and points to a potential therapeutic target for imaging markers for vascular diseases.

### Macrophage targeting strategies for CT imaging

Macrophages play a key role in the destabilization of atherosclerotic plaques and progression of AAA, through the promotion of chronic inflammation, ECM degradation, and foam cell formation 55, 103. As already mentioned, in both disease entities, macrophage phenotype plays a crucial role in disease course: pro-inflammatory type M1 macrophages are involved in maintaining inflammation and the tissue destruction; anti-inflammatory type M2 macrophages are associated with tissue repair and the stabilization of lesions. Their phagocytic activity offers a biologically tractable targeting mechanism: nanoparticles injected intravenously are naturally phagocytosed by blood monocytes and tissue-resident macrophages, allowing them to be passively targeted in areas of vascular inflammation, without receptor interaction 128. Passive uptake, however, is a measure of macrophage numbers, not their activation state, and does not differentiate M1, pro-inflammatory, from M2, reparative. This concept has been incorporated into many of the early iodine-based, as well as the more recent metal-based nanoparticles and receptor-mediated targeting contrast agents.

#### Iodine-based nanoparticles

The first macrophage-targeted CT imaging was demonstrated by Hyafil* et al.* They administered N1177 (Figure 4B) – crystalline iodinated nanoparticles dispersed with biocompatible surfactants – intravenously in a rabbit model of balloon-injury-induced aortic atherosclerosis (Figure 4A). They demonstrated that macrophage infiltration could be detected on a 64-slice clinical CT scanner 2 hours after injection (Figure 4C, a-d) 128. Plaque enhancement was significantly greater than with conventional iodinated contrast agents, iopamidol (P < 0.001). Moreover, transmission electron microscopy (TEM) and energy-dispersive spectroscopy revealed the accumulation of nanoparticles in the lysosomes of macrophages. Macrophage infiltration (> 20% of intimal area) was seen in 90% of histological sections associated with plaques exceeding the enhancement threshold. This discovery made a quantitative correlation between CT signal and macrophage load 128. The spatial association between N1177 signal and plaque localization was confirmed with color-coded density maps, co-registered with CT angiography, and no enhancement was seen in control animals (Figure 4C, e-g).

A further iodine-based approach was explored by Bhavane* et al.*, who encapsulated iodixanol within liposomal nanoparticles and demonstrated their feasibility for macrophage imaging in apolipoprotein E-deficient (*ApoE^-/-^*) mice using dual-energy CT 129. Spectral decomposition allowed iodine signal to be separated from calcified plaque deposits, and image-based cytometry confirmed liposome colocalization with approximately 2% of plaque macrophages – a small fraction reflecting the limited uptake efficiency of the non-targeted formulation, yet sufficient for CT-based plaque detection, as the authors note 129.

#### Metal-based nanoparticles

The next investigations compared the use of gold nanoparticles as an alternative to iodine, with their better X-ray attenuating capacity and design flexibility. Qin *et al.* have created a new type of gold nanoparticle-based dendrimer that is functionalized with polyethylene glycol (PEG) and is fluorescently labeled with fluorescein isothiocyanate. These particles were efficiently internalized by macrophages in vitro and enabled noninvasive, dynamic three-dimensional micro-CT imaging of macrophage burden in atherosclerotic lesions of *ApoE^-/-^* mice *in vivo.* Silver staining and CD68 immunohistochemistry confirmed that nanoparticles were accumulated within plaque macrophages 130. Another unique strategy was taken by Chhour *et al.*, which involved *ex vivo* labeling of primary monocytes with 11-mercaptoundecanoic acid-capped gold nanoparticles, allowing the monitoring of monocyte recruitment to developing plaques using micro-CT 131. Mice with gold-labeled monocytes showed a greater increase in aortic attenuation than did the control mice (Δ15.3 Hounsfield unit (HU), P = 0.002); gold nanoparticles were detected in excised plaques by TEM in the monocytes 131. This type of cell labeling enables direct visualization of the migratory behavior of circulating monocytes, unlike other passive targeting strategies. This, however, has to be achieved by *ex vivo* cell processing, which is a significant hurdle towards clinical translation. Kosuge* et al.* demonstrated *in vivo* CT imaging with intravenously injected non-targeted gold nanoparticles in two distinct disease models: a carotid ligation model in hyperlipidemic and diabetic FVB mice and an angiotensin II-induced AAA model in *ApoE^-/-^* mice 132. While CT attenuation values in the perivascular area of AAAs were significantly elevated compared to uninjected controls (P = 0.0001) (Figure 5B), the difference in the carotid atherosclerosis model did not reach statistical significance, which the authors attributed to the smaller inflammatory area in the ligated carotid artery relative to the abdominal aorta.

Toczek* et al.* extended the principle of phagocytic CT imaging to AAA, employing Exitron nano 12000 – a commercially available barium sulfate nanoparticle contrast agent – in angiotensin II-infused *ApoE^-/-^* mice 133. Following intravenous injection, delayed CT imaging at 24 hours demonstrated significant aortic wall enhancement (Figure 5C) that correlated with CD68 gene expression (r = 0.85; P < 0.001), and TEM confirmed nanoparticle accumulation within macrophages of the remodeled *adventitia*. Notably, nanoparticle-enhanced CT signal obtained within the first week of AAA induction was predictive of aneurysm diameter and survival at four weeks, directly linking early inflammatory CT signal to disease outcome 133.

#### Receptor-mediated targeting

A conceptually different approach is based on receptor overexpression on activated macrophages for active targeting, as described in the studies above. Guo* et al.* developed palladium-gold nanosheets with PEG coating (Pd@Au-PEG-FA), functionalized to target the folate receptor-β (FR-β), which is selectively upregulated on pro-inflammatory macrophages and foam cells in advanced atherosclerotic plaques 134. Histological analysis of *ApoE^-/-^* mice confirmed the co-localization of FR-β with CD68+ macrophages, with high FR-β expression associated with thin fibrous caps and large necrotic cores. There were two examples of targeting specificity. First, a competitive blocking experiment showed that pre-injection of excess folic acid reduced the artery-to-background ratio from 1.94 ± 0.19 to 1.08 ± 0.05, effectively abolishing the specific signal. Second, no significant aortic signal was observed in wild-type controls or animals injected with non-targeted nanosheets 134. *In vivo* CT imaging showed aortic wall enhancement in the bloodstream after 30 minutes, highlighting the potential for real-time CT imaging of plaque with this platform; however, the long-term persistence and specificity to background uptake in the liver and kidneys remain to be evaluated.

Together, these studies illustrate the potential of macrophage-targeted CT imaging of vascular disease using various types of nanoparticles and disease models, such as iodine-, metal-, and receptor-targeted nanoparticles. The field has moved beyond simple proof-of-concept demonstrations and is now quantifying macrophage burden *in vivo*, with direct prognostic implications, such as the ability to predict AAA outcome from early CT enhancement. However, there are still serious challenges for clinical translation, including insufficient signal sensitivity at clinically acceptable doses, the lack of approved human-use nanoparticle CT contrast agents, and the inability of passive phagocytic targeting to distinguish between M1 and M2 macrophage activation states.

### HDL-based nanoparticle CT contrast agents

A biologically distinct but mechanistically related targeting strategy exploits the natural affinity of HDL for macrophage-rich atherosclerotic plaques. HDL is an endogenous lipoprotein particle that naturally accumulates in atherosclerotic lesions during reverse cholesterol transport 135. Its affinity for macrophage-rich plaques provides a biologically relevant targeting mechanism for molecular imaging. Cormode* et al.* exploited this by replacing the hydrophobic lipid core of HDL with inorganic nanocrystals while preserving the apolipoprotein surface (Figure 6B), creating HDL-mimetic contrast agents that retain HDL's natural affinity for macrophage-rich tissue 136, 137. The gold nanocrystal variant (Au-HDL) was of particular interest for CT imaging owing to the high X-ray attenuation of gold, and macrophage specificity was confirmed *in vitro* by confocal microscopy and TEM. Preferential uptake over PEG-coated control particles was consistent with receptor-mediated internalization via SR-B1 (Scavenger Receptor Class B Type 1) and ABCA1 (ATP-Binding Cassette Transporter A1), the primary macrophage receptors for HDL (Figure 6A) 136.

In *ApoE^-/-^* mice with established atherosclerosis, intravenous administration of Au-HDL produced significant signal enhancement in the aortic wall on MRI (139 ± 25% for Au-HDL versus 35 ± 11% for PEG-coated gold nanoparticle control particles (Au-PEG) controls), while *ex vivo* micro-CT of excised aortas revealed discrete high-attenuation foci colocalizing with macrophage-rich regions at histology. These foci corresponded to sites of known plaque accumulation, such as the aortic arch and renal artery bifurcation, and their absence in Au-PEG-injected animals confirmed the preferential uptake of the HDL-mimetic nanoparticles by macrophage-rich plaque regions. (Figure 6Ca) 136.

Building on this platform, Cormode* et al.* subsequently demonstrated that Au-HDL could be combined with prototype spectral CT technology to enable simultaneous material-specific decomposition of gold, iodine, and calcium in a single scan 137. In atherosclerotic mice co-injected with Au-HDL and an iodinated vascular contrast agent, gold K-edge images presented Au-HDL accumulation in the plaque wall, while iodine images delineated the vessel lumen and calcium images identified calcified structures, providing composite information on macrophage burden, stenosis, and calcification without requiring separate acquisitions (Figure 6Cb) 137. However, the imaging system used was a single-row preclinical prototype with prohibitively long scanning times, precluding *in vivo* acquisition; spectral CT imaging was therefore performed on intact frozen whole-body specimens rather than under physiological conditions, illustrating the hardware constraints that continue to limit the clinical applicability of spectral CT-based molecular imaging 137.

Collectively, these studies demonstrate that Au-HDL nanoparticles enable targeted molecular imaging of macrophage burden in atherosclerotic plaques, with *in vivo* signal assessment achieved by MRI and material-specific plaque characterization demonstrated by *ex vivo* spectral CT. While this combined approach provides diagnostic insights into plaque composition beyond conventional anatomical CT, achieving sufficient *in vivo* CT signal sensitivity is an essential prerequisite before this Au-HDL-based imaging can be considered for clinical application.

### Elastin as a molecular target for CT imaging

Elastin fragmentation is a structurally stable and disease-specific marker of AAA growth and a potentially appropriate molecular target for imaging methods that monitor the wall instability that occurs beyond diameter measurements for AAA. Inflammatory markers and collagen are not as stable as fragmented elastin because they fluctuate with disease activity, while the latter is a stable readout of the cumulative amount of structural ECM degradation. Two studies developed anti-elastin antibody-conjugated gold nanoparticles (EL-AuNPs) that selectively bind fragmented elastin and enable quantitative micro-CT imaging due to gold's high X-ray attenuation (Figure 7A, B) 138, 139. Non-reactive immunoglobulin G-conjugated control particles produced no detectable signal even after extensive elastolysis, excluding passive sequestration as the source of the signal.

In an angiotensin II-induced AAA model in low-density lipoprotein receptor-deficient (*LDLr^-/-^*) mice, Wang* et al.* administered EL-AuNPs (designated EL-GNPs in the original publication) via retro-orbital injection. EL-AuNP accumulation at aneurysmal sites was confirmed within 24 hours by histological co-localization with fragmented elastin 138. Although *in vivo* CT imaging was not feasible at tested nanoparticle concentrations due to insufficient contrast relative to surrounding tissue, *ex vivo* micro-CT of explanted aortas clearly visualized the spatial distribution of accumulated EL-AuNPs, with markedly higher signal intensity in aneurysmal compared to healthy aortic segments (Figure 7Ca). The signal intensity demonstrated a strong correlation with *ex vivo* burst pressure (R^2^ = 0.9415), whereas neither aortic dilation (R^2^ = 0.1932) nor circumferential strain (R^2^ = 0.3918) predicted rupture strength, directly challenging the adequacy of diameter-based risk assessment 138. The maximum-intensity-projection reconstructions revealed that areas of high EL-AuNP signal correlated with the ruptures observed during burst testing (Figure 7Ca), indicating that specific areas of the aneurysmal wall may have a lower strength due to the distribution of nanoparticles.

In a separate study, Lane *et al.* used a controlled elastase perfusion model in which nanoparticles were incubated e*x vivo* at three different injury levels (0, 0.5, 10 U/mL). The *ex vivo* micro-CT was clearly able to show the increase in dose dependent accumulation of EL-AuNPs, with the gold to tissue volume ratios increasing significantly between the different groups (0.37 ± 0.3%, 4.70 ± 2.2%, and 7.56 ± 2.0%, respectively), and hyperspectral microscopy demonstrated that the AuNPs co-localized mainly with fragmented elastin in the medial layer (Figure 7Cb) 139. Importantly, delivery of nanoparticles in this model was via direct tissue incubation instead of systemic injection, and thus was conducted in a less physiologically relevant but more controlled experimental setting.

Collective analysis of these studies suggests that the elastin-targeted gold nanoparticles can be used to image degradation of the ECM in the wall of the aneurysm. These nanoparticles provide molecular imaging that is not possible with traditional imaging, in particular a strong correlation with structural weakness and rupture risk. Imaging *in vivo*, however, is still a critical step to be met before this technique can be adopted for clinical use, since both studies were performed with explanted aortas.

## Discussion

### Comparative assessment of targeting strategies

The above strategies focus on three biological aspects of vascular disease: inflammation, lipoprotein trafficking, and ECM structural degradation. Each strategy has been demonstrated to be a proof-of-concept, but there are significant differences in translational maturity and biological specificity.

Macrophage targeting is the most extensively investigated approach, encompassing the broadest range of nanoparticle platforms and the largest number of studies. Importantly, it is the only strategy in this review to have demonstrated a direct link between imaging signal and disease outcome – specifically, the finding by Toczek* et al.* 133 that early CT enhancement predicted aneurysm progression and survival at four weeks. The transition from passive iodine-based phagocytosis to receptor-mediated active targeting is aimed at increasing biological specificity, with the former being nonselective for macrophage infiltration, whereas the latter system used by Guo *et al.* 134 discriminates between activated (pro-inflammatory) and quiescent macrophages. This distinction is relevant to the disease process, since M1 and M2 macrophages play opposing roles, as described above. This is a cost in terms of the added formulation complexity, but the *ex vivo* explantation of cells by Chhour *et al.* 131 – although conceptually interesting – is not clinically translatable due to the requirement for individual *ex vivo* cell processing.

In contrast, targeting done via HDL is different. It does not involve passive accumulation or synthetic receptor ligands; instead, it uses an endogenous transport pathway with built-in macrophage affinity. The receptor-mediated uptake mechanism may offer a degree of specificity that passive phagocytosis cannot match, though this has yet to be demonstrated *in vivo*. In both available studies, CT-based imaging was restricted to excised or frozen tissue, and whether sufficient signal sensitivity can be achieved *in vivo* on a clinical scanner remains an open question 136, 137.

Elastin targeting differs from the other strategies in one important way: it addresses structural wall failure rather than inflammation. The strong correlation between EL-AuNP signal and *ex vivo* burst pressure is arguably the most clinically relevant individual finding in this entire review, as it directly challenges the adequacy of diameter-based rupture risk assessment, upon which current clinical guidelines base their recommendation for elective repair at 55 mm 89, 138. The demonstration that molecular imaging of elastin degradation outperforms diameter in predicting wall failure strength raises the prospect of a shift from size-based to biology-guided risk stratification, a clinical implication that, if validated *in vivo*, would extend well beyond the field of CT imaging. At the same time, elastin targeting is the most translationally immature of the three strategies: neither study achieved *in vivo* CT imaging, and the methodological gap between *ex vivo* proof-of-concept and *in vivo* applicability is the largest of all strategies reviewed here 138, 139.

Table 2 summarizes the nanoparticle platforms, imaging settings, key CT findings, and primary translational barriers for all studies discussed above.

Although each strategy has a different major obstacle—sensitivity of the macrophages in the case of macrophage-targeted formulations, *in vivo* validation of HDL-mimetic nanoparticles, and shifting from *ex vivo* proof-of-concept to systemic *in vivo* use for elastin-targeted agents—the pattern is the same and reflected in Table 2. What these strategies share is that imaging has been predominantly performed in small animal models using preclinical micro-CT systems, and no targeted CT contrast agent has entered clinical evaluation to date. The limiting factor is therefore not a shortage of viable biological targets, but a defined set of well-characterized, strategy-specific translational barriers.

### Translational considerations

The transition from preclinical feasibility studies to clinical implementation represents the central and as yet unresolved challenge in molecular CT imaging of vascular disease. A review of the available studies reveals several consistent barriers.

The primary and most important constraint is that of signal sensitivity. Most of the reviewed studies relied on micro-CT, a technique that offers spatial resolution sufficient to visualize the thin vessel walls of murine models but does not reflect the sensitivity constraints of clinical CT systems. The two modalities differ fundamentally across several parameters relevant to molecular imaging: micro-CT systems employ micro-focus X-ray sources with spatial resolutions of 10–100 µm and flat-panel detectors with pixel sizes below 150 µm, whereas clinical CT systems achieve resolutions in the submillimeter range with curved detector arrays optimized for large-field, low-dose acquisition 140. Radiation doses in preclinical micro-CT are substantially higher than those acceptable in clinical practice, and acquisition times are considerably longer, collectively yielding signal-to-noise ratios that cannot be reproduced under clinical conditions 140, 141. As a consequence, signal thresholds demonstrated in micro-CT studies cannot be directly extrapolated to clinical scanners. The absence of detectable contrast enhancement on a micro-CT system, as reported by Wang* et al.* with EL-AuNPs at tested concentrations, should therefore be interpreted as evidence of a current sensitivity gap, not as proof of fundamental clinical infeasibility 138. Kosuge* et al.* illustrated this directly: in the carotid ligation model, the volume of inflammatory tissue was too small relative to the CT voxel size to produce a detectable signal difference - a limitation the authors attributed to the smaller lesion size compared to the aorta, where the same agent generated a significant signal 132.

These findings show an important consideration throughout the review. The feasibility of molecular CT imaging varies across the vascular system and depends on three interacting factors: vessel size, wall thickness, and motion. Vessel size and wall thickness together influence the severity of the partial volume effect, an inherent limitation of voxel-based CT: when the vessel wall is small relative to the CT voxel, its attenuation is averaged with that of the adjacent luminal blood and perivascular tissue, diluting the measurable contrast and raising the local concentration required for detection. The abdominal aorta, with the largest diameter and thickest wall, is the least affected and therefore offers the most favorable ratio of diseased to healthy tissue within a voxel, whereas the thinner walls of the carotid and coronary arteries are far more susceptible to partial volume averaging. Motion imposes a further, territory-specific penalty: the coronary arteries undergo continuous cardiac motion, compounded by respiratory excursion, that necessitates electrocardiogram (ECG)-gated acquisition and high temporal resolution 142. The carotid arteries are superficial and largely free of cardiac motion but small in caliber, whereas the abdominal aorta is comparatively static. Although the coronary territory is a principal site of clinical interest in atherosclerosis, it represents the least favorable combination of all three factors, whereas the abdominal aorta represents the most favorable; the imaging advantage of AAA studies is therefore structural, independent of the agent used.

Against this background, the finding by Hyafil* et al.* – detection of macrophage-dependent plaque accumulation on a clinical 64-slice scanner – represents the most translationally relevant technical achievement within this review, one that has not been replicated by any subsequently developed agents 128. However, as Hyafil* et al.* employed a rabbit rather than a mouse model, this result also benefits from the larger vessel wall of the model organism and does not provide direct evidence for feasibility in humans.

Beyond the sensitivity gap between preclinical and clinical systems, detection capability also depends on the CT platform itself. On conventional energy-integrating scanners, iodinated contrast is detectable only at comparatively high concentrations, and the measured attenuation cannot be assigned to a specific element, so a nanoparticle label cannot be distinguished from vascular calcium or luminal blood. Dual-energy and spectral CT address the latter limitation through material decomposition, which separates the contrast material from calcium. Photon-counting detector CT (PCD-CT) extends this further: by resolving the energy of individual photons, it enables element-specific K-edge imaging and provides higher contrast-to-noise ratios 143 and spatial resolution at matched dose 144. Si-Mohamed* et al.* demonstrated the *in vivo* value of this capability, using gold-nanoparticle K-edge imaging to separate the macrophage-associated probe signal from iodine and calcium and to track plaque macrophage burden far more closely than conventional CT (r = 0.82 vs 0.41) 145. The gain, however, is primarily one of specificity rather than raw sensitivity: the minimum detectable concentration on PCD-CT is still on the order of 1 mg I/mL and rises as the target shrinks (0.5 and 1.0 mg I/mL for 8-mm and 4-mm lesions, respectively) 146, so the requirement for relatively high local agent concentrations persists. The recent clinical introduction of PCD-CT and ongoing detector improvements may therefore narrow, but are unlikely to eliminate, the sensitivity bottleneck that currently constrains molecular CT.

A second barrier concerns targeting efficiency. Even when nanoparticle accumulation within the target tissue has been demonstrated, it remains unclear what fraction of the relevant cell population is actually reached. Bhavane *et al.* showed, using image-based cytometry, that their liposomal nanoparticles colocalized with only ~ 2% of plaque macrophages 129. The fact that a CT signal was still detectable can be explained by the high iodine payload per particle - a finding that simultaneously illustrates how narrow the signal margin is and how little room remains for further reductions in uptake efficiency. Bhavane *et al.* did not report the effective iodine concentration within the plaque, which precludes a reliable assessment of whether the achieved local accumulation exceeded the detection limits of clinical CT systems. Depending on the scanner platform and object size, these detection limits range from 0.02 to 0.55 mg/mL 129, 147. In passive uptake mechanisms, this efficiency remains strongly influenced by local factors, including macrophage density, perfusion, and tissue permeability, all of which vary considerably across animal models and human disease. The biological specificity of receptor-based targeting, such as with nanoparticles targeted to FR-β developed by Guo *et al.* 134, is a way to solve the leveraging challenge. Although they demonstrated phantom linearity of 122.72 HU per mg/mL 134 and measured *in vivo* contrast agent concentrations in the blood, Guo *et al.* did not report *in vivo* concentrations within the plaque. It remains unclear whether this will lead to increased uptake efficiency *in vivo*, since there is no report comparing the *in vivo* uptake efficiency of the two approaches under the same experimental conditions. The in-tissue measurements of the contrast agents should be reported in the future in a consistent manner, allowing for comparison with known clinical detection limits.

The third barrier has to do with off-target accumulation and safety. Toczek *et al.* observed in vitro that their nanoparticles were taken up not only by macrophages but also by endothelial cells and smooth muscle cells - a finding which questions the selectivity of passive targeting approaches 133. In addition, the number of signals within the mononuclear phagocyte system decreased slightly over 6 days, suggesting the signals lingered in the tissues 133. For clinical use, detailed biodistribution and clearance information is critical but none of the reviewed studies provide these details.

In addition to these obstacles, there are two additional factors that are not discussed in the studies reviewed which have implications for clinical translation. Dosimetry is the first consideration. Local concentrations of contrast agents are much higher in CT than in MRI or nuclear imaging to produce a detectable signal 148. High systemic exposure is associated with the required tissue concentrations. To date, preclinical blood-pool CT imaging in mice has been performed with gold doses of around 0.5 g Au/kg body weight, corresponding to about 2.5 µmol Au/g. Such dosing elicits crucial questions regarding long-term tissue retention, biodegradability, and the acceptability of these doses for regulatory approval in diagnostic applications 149. The second is related to manufacturing. The more complex formulations containing targeted nanoparticles are problematic in terms of good manufacturing practice (GMP) compliant production, batch-to-batch reproducibility, and stability, aspects which are generally required for regulatory approval but are not reported in any of the reviewed studies 150, 151.

The translational barriers outlined in this review are linked together in a consistent way: so far, none of the studies were able to tackle simultaneously the issues of signal sensitivity, targeting efficiency, off-target accumulation, safety profile, dosimetry, and end up with a feasible manufacture. The current work offers proof-of-concept data of the biological targeting strategies, but they are not optimized systematically like would be needed for clinical testing.

### Untranslated molecular targets

In addition to the three strategies reviewed here, a thorough search of the molecular imaging literature shows a number of other promising, biologically relevant targets that have been validated *in vivo* in the context of MRI, PET or US, but which have no CT-based equivalent.

Gadolinium-based MRI probes such as P947 can detect MMP activity *in vivo* during experimental AAA 152 and more recently ⁶⁴Cu-RYM2 153 which also exhibited specific binding to human aortic tissue in addition to MMP activity. VCAM-1 is expressed on activated endothelium at early stages of inflammatory cascade, and VCAM-1-directed nanoparticles have been used for MRI based contrast enhancement of atherosclerosis in mice 154 and VCAM-1-directed microbubbles 155. The αvβ3-integrin is overexpressed during plaque-associated neovascularization and has been targeted for radionuclide imaging, but there is no report of an equivalent for imaging by CT 156. The presence of active microcalcification within the fibrous cap is another biologically important process: ¹⁸F-NaF PET identifies sites of active calcification in coronary atherosclerosis, and shows that ¹⁸F-NaF tracer uptake does not simply reflect the total calcium burden 157, indicating that the biological process of calcium and the anatomical substrate are different entities, and that conventional CT can not detect both. This strategy has been tested in patients with coronary and carotid atherosclerosis, proving clinical proof-of-concept of the validity of active microcalcification as an imaging biomarker of plaque vulnerability independent of the calcium burden 158. Fibrin, which is involved in endothelial disruption and thrombosis, is a target for which gold nanoparticles can be used to specifically target acute thrombosis, but has yet to be applied in chronic vascular wall pathology with CT 159. Ultrasmall superparamagnetic iron oxide (USPIO) enhanced MRI has also been used to image macrophage infiltration of patients with carotid atherosclerosis, where measurable inflammation of the plaque and its response to lipid-lowering therapy have been reported 160.

The absence of CT-compatible probes for these targets reflects constraints that go beyond the general translational barriers discussed above. The millimolar concentrations required for CT signal generation are difficult to achieve for targets that are not amenable to bulk cellular accumulation, in contrast to the macrophage phagocytosis mechanism that underpins the strategies reviewed here 148. PET and MRI circumvent this through intrinsically higher detection sensitivity, operating at picomolar and micromolar concentrations respectively 148. Active microcalcification differs in this respect: here the limiting factor is not achievable concentration but biological specificity, as conventional CT measures static calcium density and cannot resolve the active mineralization process that ¹⁸F-NaF PET detects 157.

### Further perspectives

Among the untranslated targets discussed above, MMP activity represents a tractable translational opportunity. As a direct readout of the proteolytic state of the vessel wall, it is pathologically relevant in both fibrous cap degradation and AAA wall failure, and it is *in vivo* imaging feasibility has already been established with both MRI and PET probes, leaving the development of a CT-compatible equivalent as the remaining translational step.

Ultimately, the role of molecular CT is best understood not as a replacement for the more sensitive MRI and nuclear techniques, but as a means of extending biological characterization of the vessel wall to the cross-sectional modality most widely used in routine vascular assessment, a prospect contingent on advances in probe design and on the translational progress outlined above.

## Conclusion

The reviewed studies showed that CT can detect biologically relevant processes in atherosclerotic and aneurysmal vessel walls, such as macrophage infiltration, lipoprotein trafficking, and structural ECM degradation, providing proof of the feasibility of using CT as a platform for molecular imaging, not just for its anatomical applications. The potential is translated into the clinic but requires systematic validation in large-animal models (*in vivo* validation of HDL mimetics, *ex vivo*-to-*in vivo* transition of elastin-targeted agents, signal sensitivity for macrophage-targeted agents) to enable meaningful assessment of human feasibility. The underlying biological basis for molecular CT imaging of vascular disease is solid, but translating these concepts into reality is still in the future.

## Figures and Tables

**Figure 1:**
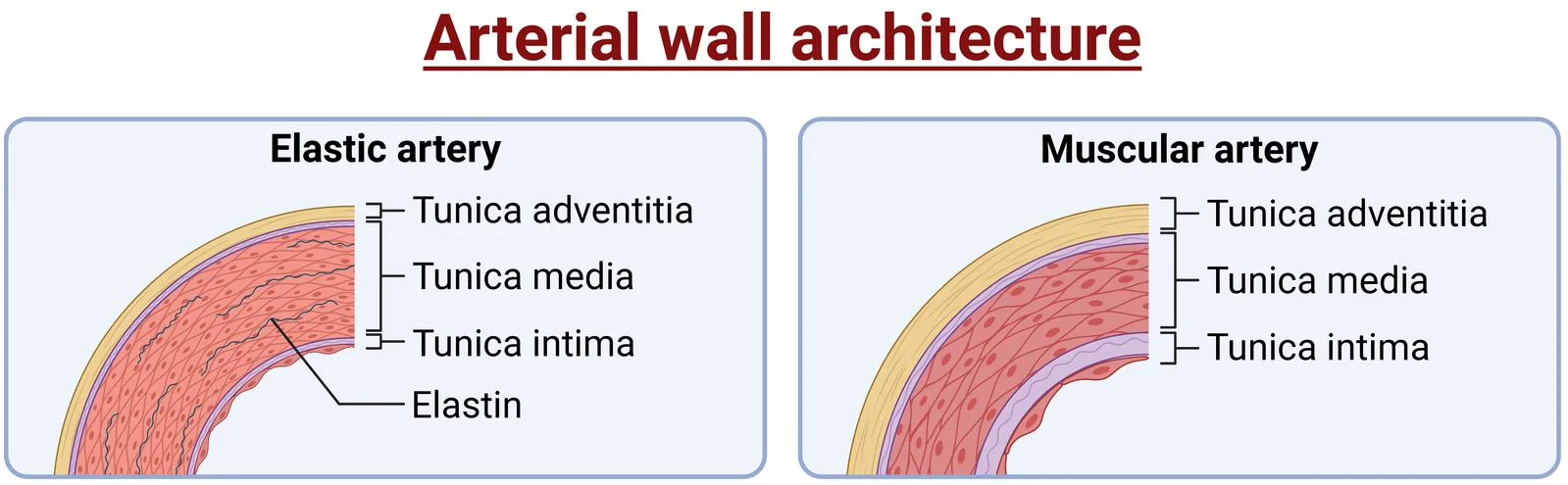
** Arterial wall architecture.** Schematic cross sections of an elastic artery (left) and a muscular artery (right), showing the three-layered structure of the wall, *tunica intima*, *tunica media*, and *tunica adventitia*. Elastic arteries have a thick media that is rich in elastin, while muscular arteries have a greater percentage of VSMCs in the media. Figure created with BioRender.com.

**Figure 2:**
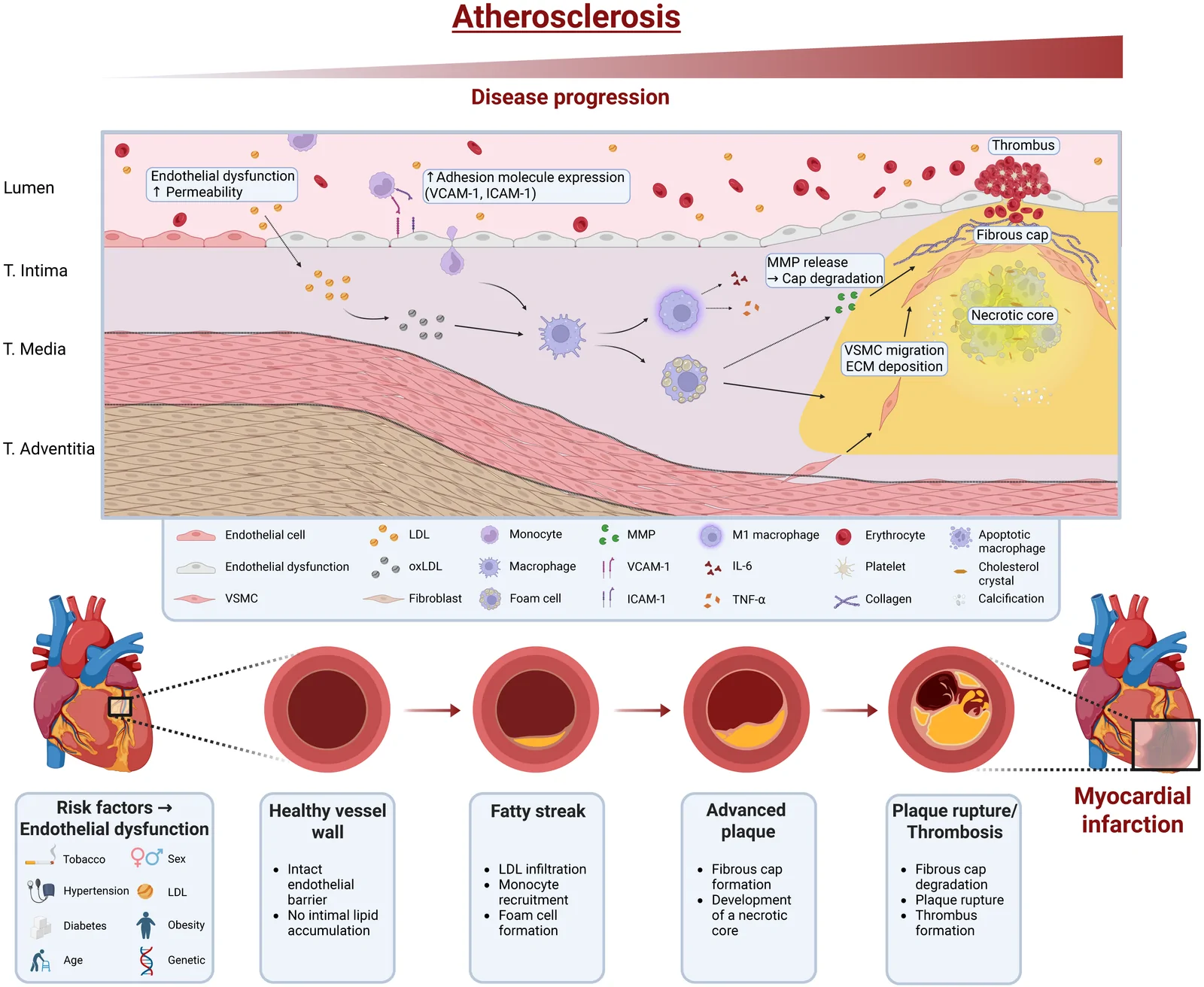
** Atherosclerosis disease progression.** Endothelial dysfunction promotes LDL infiltration and oxidation, inducing adhesion molecule expression (VCAM-1, ICAM-1) and monocyte recruitment. Monocytes differentiate into macrophages and lipid-laden foam cells, which, together with VSMC migration, ECM deposition, fibrous cap formation, necrotic core development, and calcification, drive plaque progression. MMP-mediated cap degradation can result in plaque destabilization, rupture, thrombosis, and myocardial infarction. Abbreviations: ECM: extracellular matrix; ICAM-1: intercellular adhesion molecule 1; IL-6: interleukin-6; LDL: low-density lipoprotein; MMP: matrix metalloproteinase; oxLDL: oxidized low-density lipoprotein; T.: *Tunica*; TNF-α: tumor necrosis factor alpha; VCAM-1: vascular cell adhesion molecule 1; VSMC: vascular smooth muscle cell. Based on 59-61. Figure created with BioRender.com.

**Figure 3:**
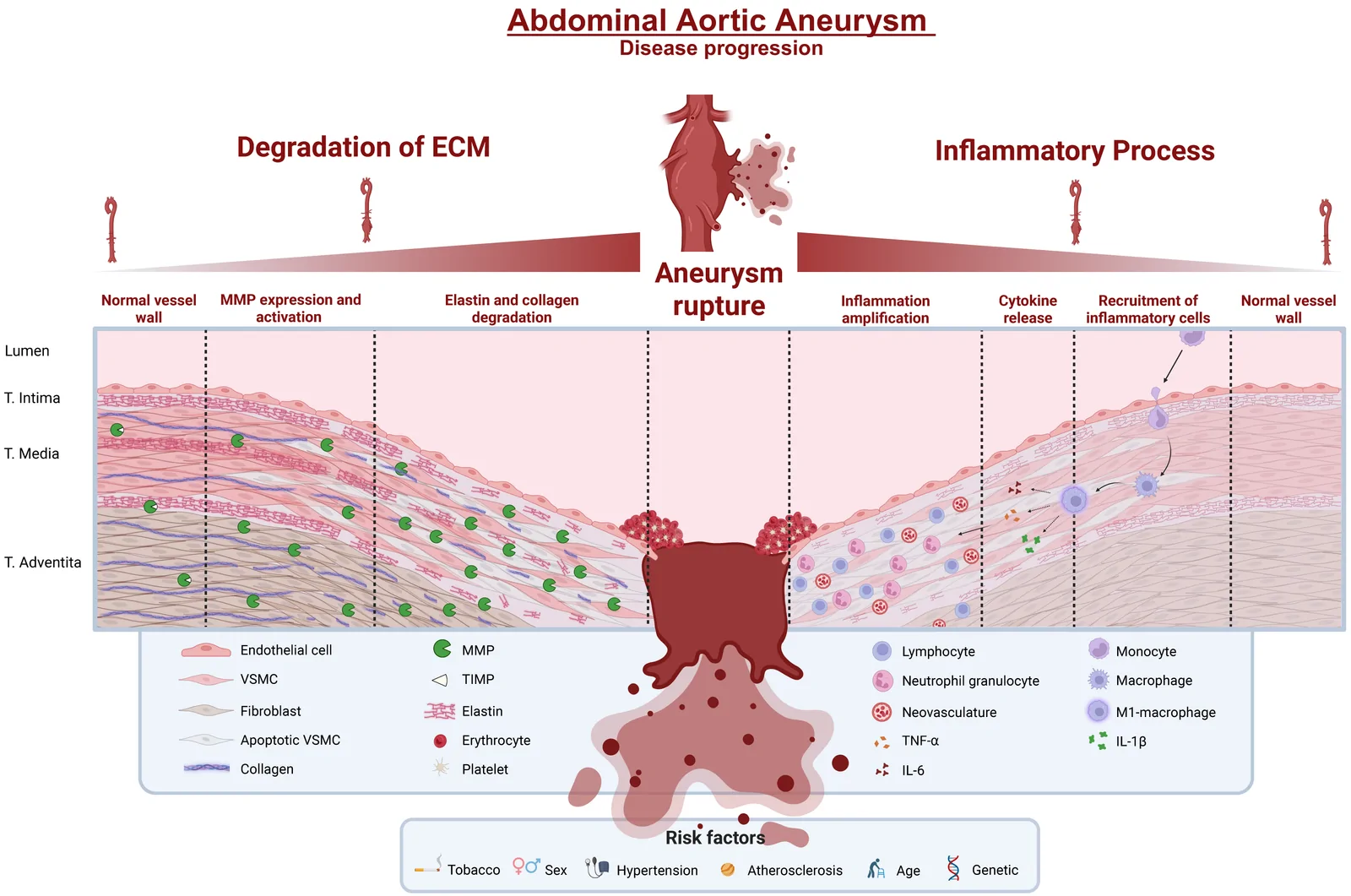
** AAA disease progression.** Chronic inflammation and protease activation drive ECM degradation within the aortic wall. Increased expression of MMPs promotes fragmentation of elastin and collagen, leading to medial weakening and VSMC apoptosis. Chemokine-mediated recruitment of monocytes, lymphocytes, and neutrophils amplifies inflammation by releasing cytokines such as TNF-α, IL-1β, and IL-6. Neovascularization further facilitates immune cell infiltration. Progressive wall destabilization ultimately results in aneurysm expansion and rupture. Abbreviations: ECM: extracellular matrix; IL-1β: interleukin-1 beta; IL-6: interleukin-6; MMP: matrix metalloproteinase; T.: *Tunica*; TIMP: tissue inhibitor of metalloproteinases; TNF-α: tumor necrosis factor alpha; VSMC: vascular smooth muscle cell. Figure created with BioRender.com based on 112, 114, 115.

**Figure 4:**
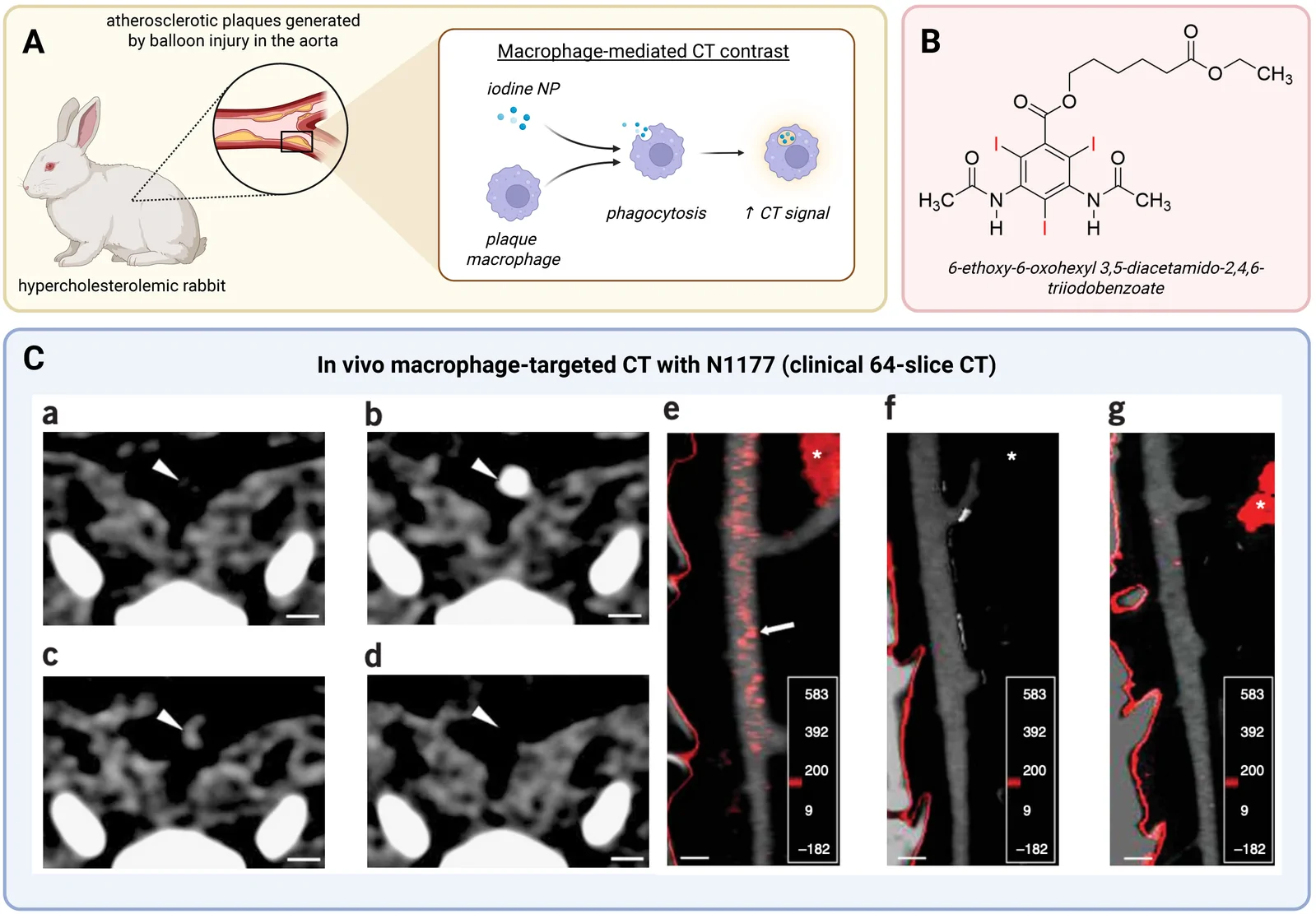
** Iodinated nanoparticle CT imaging of plaque macrophages (N1177).** (A) Targeting mechanism: in hypercholesterolemic rabbits with balloon-injury aortic plaques, iodinated nanoparticles (iodine NP) are phagocytosed by plaque macrophages, increasing CT signal. (B) N1177 iodinated compound: 6-ethoxy-6-oxohexyl 3,5-diacetamido-2,4,6-triiodobenzoate (C₁₉H₂₃I₃N₂O₆), an esterified diatrizoate derivative (three iodine atoms in red); its low aqueous solubility yields surfactant-stabilized nanoparticles (~259 nm) taken up by macrophages. (C) In vivo macrophage-targeted CT with N1177 (clinical 64-slice scanner): axial CT of the same plaque (arrowheads) before, during, and 2 h after N1177 (a–c) or a conventional agent (d), with enhancement after N1177 only; attenuation-coded regions fused to the aortic angiogram show intense red plaque signal with N1177 (e), absent with the conventional agent (f) and in a control rabbit (g). Plaque density rose 29.7 ± 6.0 → 43.0 ± 7.3 HU (net 13.3 ± 1.0 vs 4.1 ± 0.9 HU, P < 0.001). Arrowhead, plaque; arrow, plaque enhancement; asterisk, spleen; color scale, HU (-182 to 583); scale bar, 5 mm. Figure created with BioRender.com. Panel (C) adapted with permission from 128, copyright 2007 Springer Nature. The chemical structure in (B) was drawn by the authors using ChemDraw. CT, computed tomography; HU, Hounsfield units; NP, nanoparticle.

**Figure 5:**
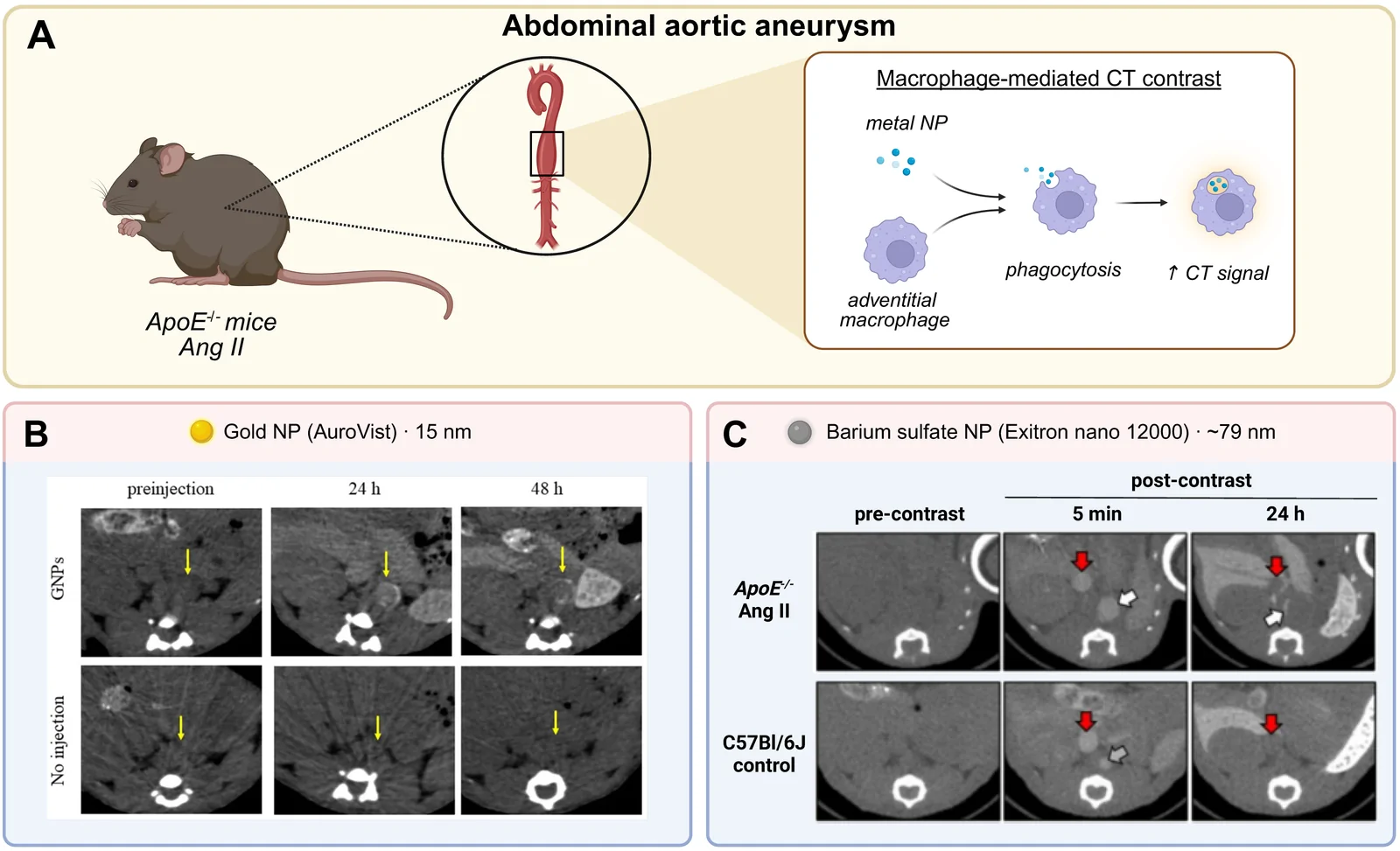
** Metal-based nanoparticles for macrophage-mediated CT imaging of AAA.** (A) Targeting mechanism: in the ApoE⁻ᐟ⁻ + Ang II model, metal nanoparticles are phagocytosed by adventitial macrophages, increasing CT signal. Created with BioRender.com. (B) Gold nanoparticles (AuroVist, 15 nm): perivascular CT attenuation rises after injection (GNPs) but not in uninjected controls (No injection), at preinjection/24 h/48 h (P = 0.0001); yellow arrow, perivascular AAA. Adapted with permission from 132, copyright 2021 the authors, CC BY 4.0. (C) Barium sulfate nanoparticles (Exitron nano 12000, ~79 nm): after blood-pool clearance the aneurysm wall opacifies at 24 h in ApoE⁻ᐟ⁻ + Ang II mice but not in C57Bl/6J controls (pre-contrast/5 min/24 h; enhancement in the hundreds of HU, CD68-correlated, r = 0.85). White arrow, AAA; red, inferior vena cava; grey, aorta. Adapted with permission from 133, copyright 2021 the authors, CC BY 4.0. Figure created with BioRender.com. AAA, abdominal aortic aneurysm; Ang II, angiotensin II; ApoE⁻ᐟ⁻, apolipoprotein E-deficient; CT, computed tomography; GNP, gold nanoparticle; HU, Hounsfield unit; NP, nanoparticle.

**Figure 6:**
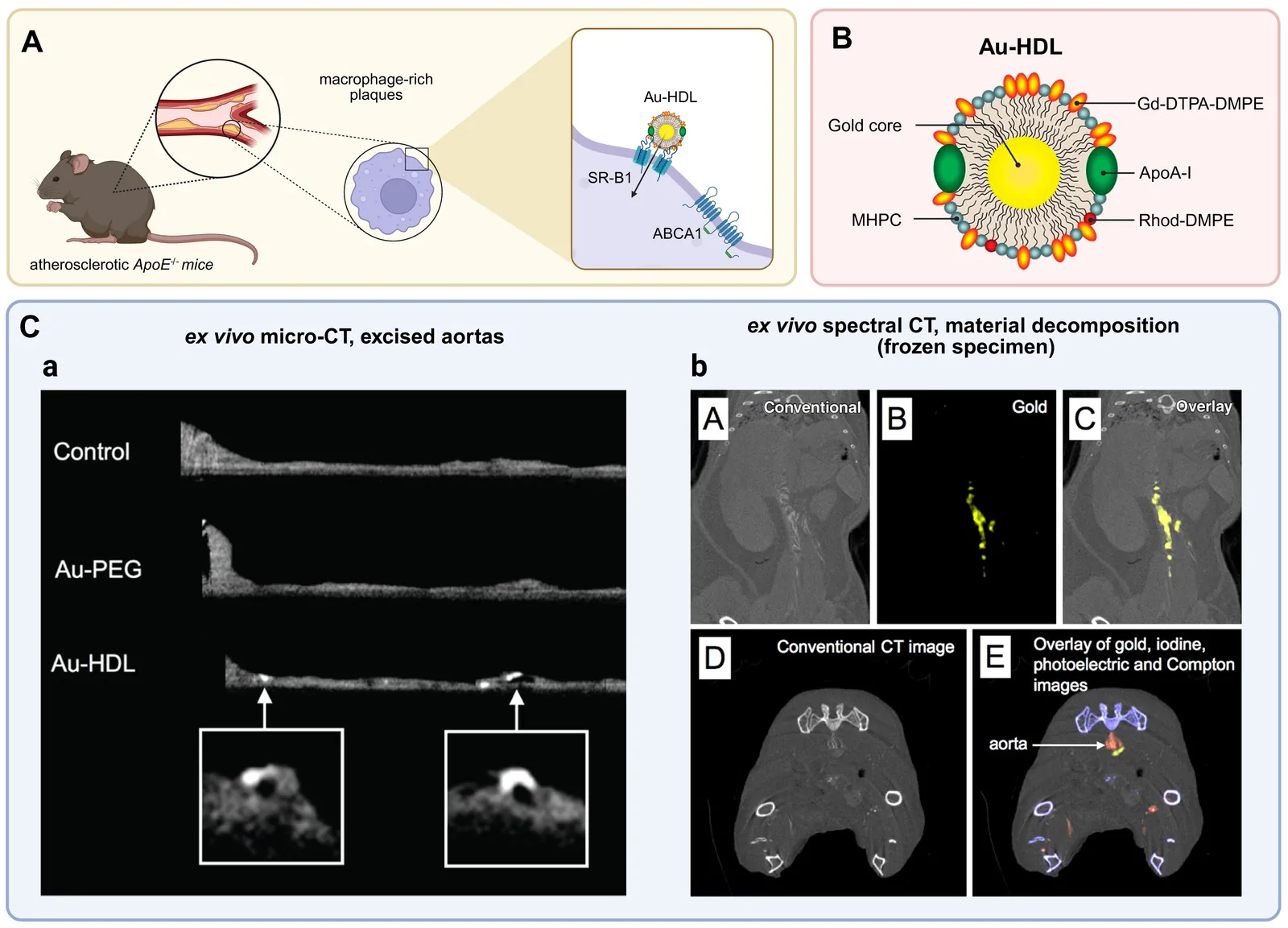
** Au-HDL for molecular CT imaging of plaque macrophages.** (A) Targeting mechanism: in ApoE⁻ᐟ⁻ plaques, Au-HDL is taken up by macrophages via SR-B1 (ABCA1 shown for context). (B) Au-HDL architecture: gold core, phospholipid monolayer (MHPC, Gd-DTPA-DMPE, Rhod-DMPE), and apoA-I. (C) Representative CT imaging. Left, ex vivo micro-CT: selective Au-HDL accumulation in macrophage-rich plaque, absent with Au-PEG and control. Right, ex vivo spectral CT (frozen specimen): plaque-specific Au-HDL (gold), distinct from the iodine-filled lumen. Figure created with BioRender.com. Panel (B and Ca) adapted with permission from 136, copyright 2008 American Chemical Society. Panel (Cb) adapted with permission from 137, copyright 2010 Radiological Society of North America. ABCA1, ATP-binding cassette transporter A1; apoA-I, apolipoprotein A-I; ApoE⁻ᐟ⁻, apolipoprotein E-deficient; Au-HDL, gold-core high-density lipoprotein nanoparticle; Au-PEG, PEGylated gold nanoparticle; CT, computed tomography; Gd-DTPA-DMPE, gadolinium-DTPA-dimyristoylphosphatidylethanolamine; MHPC, 1-myristoyl-2-hydroxy-sn-glycero-3-phosphocholine; PEG, polyethylene glycol; Rhod-DMPE, rhodamine-dimyristoylphosphatidylethanolamine; SR-B1, scavenger receptor class B type 1.

**Figure 7:**
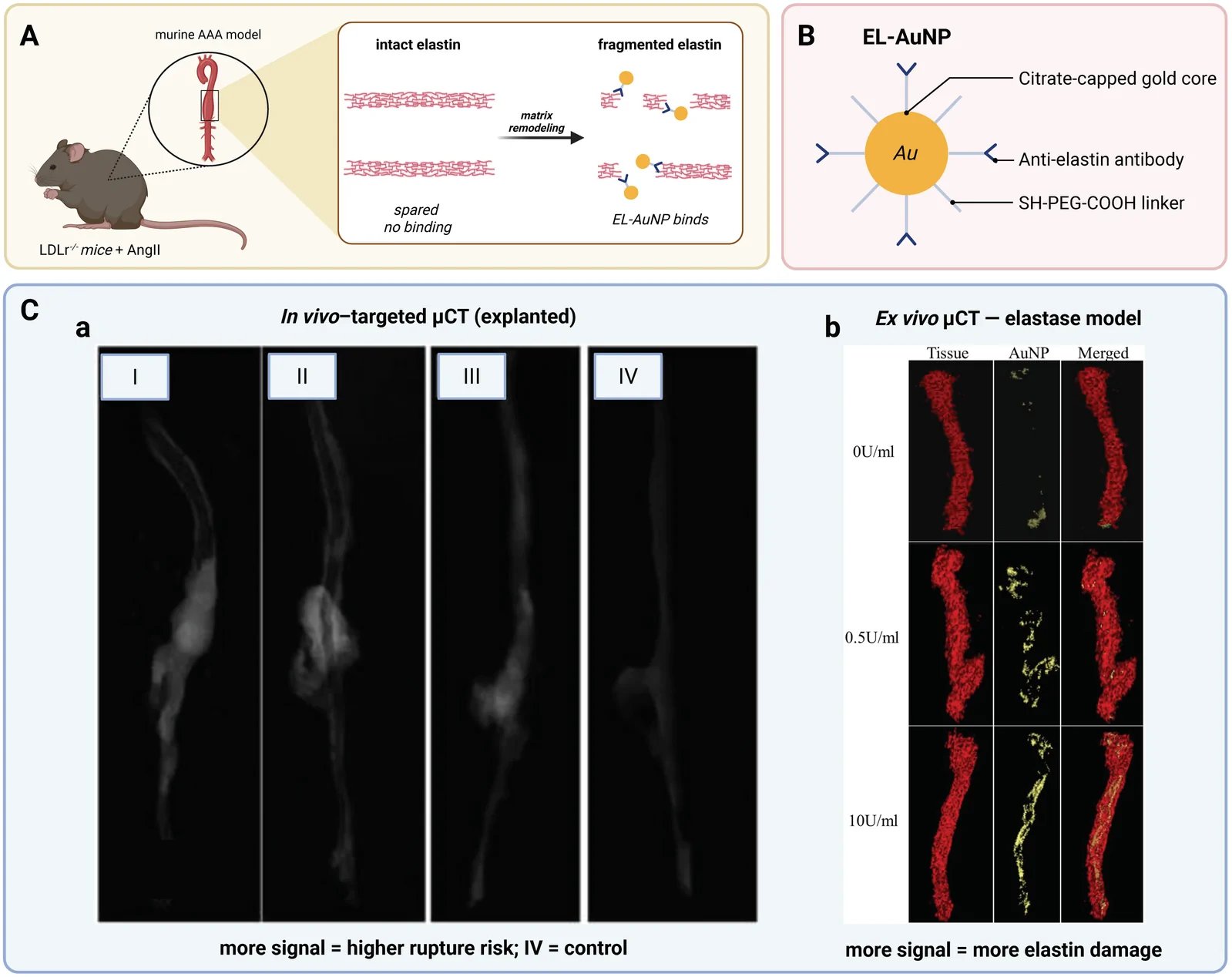
** EL-AuNP for molecular CT imaging of fragmented elastin in AAA.** (A) Targeting mechanism: matrix remodeling fragments aortic elastin, exposing neo-epitopes; EL-AuNP binds the fragmented elastin while sparing intact fibers. (B) EL-AuNP architecture: citrate-capped gold core, SH-PEG-COOH linker, and anti-elastin antibody (termed EL-GNP in 138). (C) Representative CT imaging. Left (Ca), in vivo–targeted micro-CT of explanted aortas after systemic EL-AuNP administration (LDLr⁻ᐟ⁻ + Ang II model): accumulation increases as burst pressure falls (I–III, higher rupture risk; signal–burst-pressure correlation R² = 0.94), with no signal in the age-matched control (IV). Adapted with permission from 138, copyright 2019 Ivyspring International Publisher, CC BY-NC. Right, ex vivo micro-CT (tissue and gold filtered) of elastase-perfused aortas: EL-AuNP accumulation increases with elastase dose (0/0.5/10 U/mL), tracking elastin damage. Adapted with permission from 139, copyright 2020 Biomedical Engineering Society. Figure created with BioRender.com. AAA, abdominal aortic aneurysm; Ang II, angiotensin II; Au, gold; AuNP, gold nanoparticle; CT, computed tomography; EL-AuNP, elastin-targeted gold nanoparticle; LDLr⁻ᐟ⁻, low-density lipoprotein receptor-deficient; SH-PEG-COOH, thiol-polyethylene glycol-carboxyl linker.

**Table 1:** Composition and function of arterial wall layers

Layer	Composition	Main function	Ref.
Tunica intima	Endothelial cells with surface glycocalyx basal lamina internal elastic lamina	Barrier function regulation of vascular tone, permeability, coagulation, and inflammation	26, 28-30
Tunica media	VSMCs embedded in elastin- and collagen-rich ECM elastic lamellae	Provides elasticity and compliance enables vasoconstriction and vasodilation structural support	26-28, 31, 32
Tunica adventitia	Fibroblasts in collagen- and elastin-rich ECM nerves vasa vasorum	Mechanical stability, vascular repair, and signaling	26, 27, 33, 34

VSMCs: vascular smooth muscle cells; ECM: extracellular matrix

**Table 2:** Nanoparticle-based CT contrast agents for molecular imaging of vascular disease: study characteristics and translational barriers.

Nanoparticle type	Target	Modality	Setting	Key CT result	Translational barrier	Ref.
Crystalline iodinated NPs (N1177)	Macrophage uptake (passive)	Clinical CT (64-slice)	*In vivo* (New Zealand white rabbit)	Plaque ΔHU after N1177: 13.3 vs. 4.1 HU conventional agent (P<0.001); plaques exceeding threshold showed macrophage infiltration >20% in 90% of sections	Radiation exposure; signal overlap with calcifications; optimal dosing requires further validation	130
Liposomal iodinated NPs (iodixanol)	Macrophage uptake (passive)	Micro-CT (dual-energy)	*In vivo* (*ApoE^-/-^* mouse)	Dual-energy spectral decomposition separated liposomal iodine from calcifications; liposomes colocalized with ~ 2% of plaque macrophages	No HU calibration; 40 kVp protocol not available on clinical scanners; potential hypersensitivity from DPPG surface charge	131
Dendrimer-entrapped gold NPs	Macrophage uptake (passive)	Micro-CT	*In vivo* (*ApoE^-/-^* mouse)	CT values of atherosclerotic vessels significantly elevated up to 6 h post-injection; confirmed by silver staining and CD68	No quantitative plaque delta-HU; biodistribution evaluated only up to 6 h	132
Gold NPs, *ex vivo* monocyte labeling	Monocyte recruitment	Micro-CT	*In vivo* (*ApoE^-/-^* mouse)	Aortic attenuation significantly increased vs. controls (Δ15.3 HU; P = 0.002); TEM confirmed gold NPs in plaque monocytes	*Ex vivo* cell processing not clinically translatable; increased gold loading per cell required to detect small reductions in monocyte uptake (<30%)	133
Unmodified gold NPs	Macrophage uptake (passive)	Micro-CT	*In vivo* (*ApoE^-/-^*mouse)	Significant perivascular enhancement in AAA (P = 0.0001); no significant difference in carotid model due to small lesion volume relative to voxel size	Signal insufficient in smaller lesions relative to CT voxel size; detection model-dependent	134
Barium sulfate NPs (Exitron nano 12000)	Macrophage uptake (passive)	Micro-CT	*In vivo* (*ApoE^-/-^* mouse)	Aortic wall enhancement correlated with CD68 expression (r = 0.85; P < 0.001); early CT signal predicted aneurysm diameter and survival at 4 weeks	Off-target uptake in endothelial and smooth muscle cells; prolonged tissue retention; preclinical use only	135
Pd@Au nanosheets (PEG/FA-functionalized)	FR-β on pro-inflammatory macrophages	Micro-CT	*In vivo* (*ApoE^-/-^*mouse)	CT enhancement of aortic wall visible at 30 min post-injection; FR-β co-localized with CD68 in high-risk plaques	CT signal not quantified; no delta-HU reported; high background in kidneys	136
Gold nanocrystal-core HDL-mimetic NPs	HDL receptor-mediated uptake (SR-B1 / ABCA1)	Micro-CT	*Ex vivo* (*ApoE^-/-^* mouse)	High-attenuation foci at aortic arch and renal arteries in Au-HDL aortas; absent in Au-PEG and saline controls	Low CT sensitivity; imaging restricted to excised aortas	138
Gold nanocrystal-core HDL-mimetic NPs	HDL receptor-mediated uptake (SR-B1 / ABCA1	Spectral-CT (preclinical)	*Ex vivo* (*ApoE^-/-^* mouse)	Au-HDL accumulation in aorta detected by gold K-edge images; simultaneous material decomposition of gold, iodine, and calcium in single scan	Preclinical single-row prototype; *in vivo* CT acquisition not feasible	139
Anti-elastin antibody-conjugated gold NPs	Degraded elastin in AAA wall	Micro-CT	*Ex vivo* (*LDLr^-/-^* mouse)	Higher signal intensity in aneurysmal vs. healthy aortic segments; micro-CT signal correlated with burst pressure (R²=0.9415); maximum intensity projection foci corresponded to rupture sites	*In vivo* CT signal insufficient at tested concentrations; imaging restricted to explanted aortas	140
Anti-elastin antibody-conjugated gold NPs	Degraded elastin in AAA wall	Micro-CT	*Ex vivo*, elastase perfusion model (graded injury)	Dose-dependent micro-CT signal (Gold-to-tissue ratios 0.37%, 4.70%, 7.56% at 0, 0.5, 10 U/mL); signal correlated with lumen area compliance and percent dilation	*Ex vivo* tissue incubation only; no systemic delivery; dilation below clinical AAA threshold	141

AAA: abdominal aortic aneurysm; ABCA1: ATP-binding cassette transporter A1; *ApoE^-/-^*: apolipoprotein E–deficient; Au-HDL: gold nanocrystal high-density lipoprotein; Au-PEG: gold polyethylene glycol; CT: computed tomography; DPPG: 1,2-dipalmitoyl-sn-glycero-3-phosphoglycerol, sodium salt; FR-β: folate receptor β; HU: Hounsfield unit; *LDLr^-/-^*: low-density lipoprotein receptor-deficient; NPs: nanoparticles; SR-B1: scavenger receptor class B type 1; TEM: transmission electron microscopy.

## Abbreviations

AAA: abdominal aortic aneurysm
ABCA1: ATP-Binding Cassette Transporter A1
*ApoE^-/-^*: apolipoprotein E-deficient
Au-HDL: gold nanocrystal high-density lipoprotein
Au-PEG: gold polyethylene glycol
CT: computed tomography
CTA: computed tomography angiography
CVD: cardiovascular disease(s)
ECG: electrocardiogram
ECM: extracellular matrix
EL-AuNPs: anti-elastin antibody-conjugated gold nanoparticles
FR-β: folate receptor-β
GMP: good manufacturing practice
HDL: high-density lipoprotein
HU: Hounsfield unit
ICAM-1: intercellular adhesion molecule-1
IL-1β: interleukin-1β
IL-6: interleukin-6
LDL: low-density lipoprotein
*LDLr^-/-^*: low-density lipoprotein receptor-deficient
MMP: matrix metalloproteinase
MRI: magnetic resonance imaging
NP: nanoparticle
oxLDL: oxidized low-density lipoprotein
PCD-CT: Photon-counting detector computed tomography
Pd@Au-PEG-FA: folate-conjugated palladium-gold nanosheets with polyethylene glycol coating
PEG: polyethylene glycol
PET: positron emission tomography
SPECT: single-photon emission computed tomography
SR-B1: Scavenger Receptor Class B Type 1
TEM: transmission electron microscopy
TIMP: tissue inhibitor of metalloproteinase
TNF-α: tumor necrosis factor-α
US: ultrasound
USPIO: ultrasmall superparamagnetic iron oxide
VCAM-1: vascular cell adhesion molecule-1
VSMC: vascular smooth muscle cell
